# Why Did Treg and Immune Tolerance Win Nobel Prize This Year?

**DOI:** 10.1093/gpbjnl/qzaf124

**Published:** 2025-12-12

**Authors:** Song Guo Zheng

**Affiliations:** Department of Immunology, School of Cell and Gene Therapy / Songjiang Research Institute / Songjiang Hospital, Shanghai Jiao Tong University School of Medicine, Shanghai 201600, China

From the 1995 identification of CD4^+^CD25^+^ regulatory T (Treg) cells by Shimon Sakaguchi to the 2025 tolling of Stockholm’s bells, this “minority population” has moved to center stage in immune tolerance. Recounted here from a first-hand perspective are the pivotal milestones in the Treg chronicle. Each experiment addressed a single question: why does the immune system refrain from attacking self? In jointly honoring the discoverers of Treg cells (Shimon Sakaguchi) and their master regulator Foxp3 (Mary E. Brunkow and Fred Ramsdell), the Nobel Committee not only celebrates a scientific saga but also proclaims that mastering Treg cells equips us with a universal remote to treat autoimmunity, transplant rejection, and even tumor immune evasion.

I still remember that during a Cold Spring Harbor conference in 2018, Dr. Sakaguchi and I had a dinner. During the dinner, I asked him if Treg cells could possibly win the Nobel Prize. Shimon shook his head firmly, stating that it was impossible. I didn’t fully agree with his judgment at the time and told him that Treg cells might be favored by the Nobel Committee, because although Treg cells are not numerous, their functions are extremely important. In fact, we know each other very well and meet together almost every year (**Figure 1**). He is a very humble person and is unwilling to make overly optimistic predictions. Seven years later, my prediction has indeed come true!

**Figure 1:**
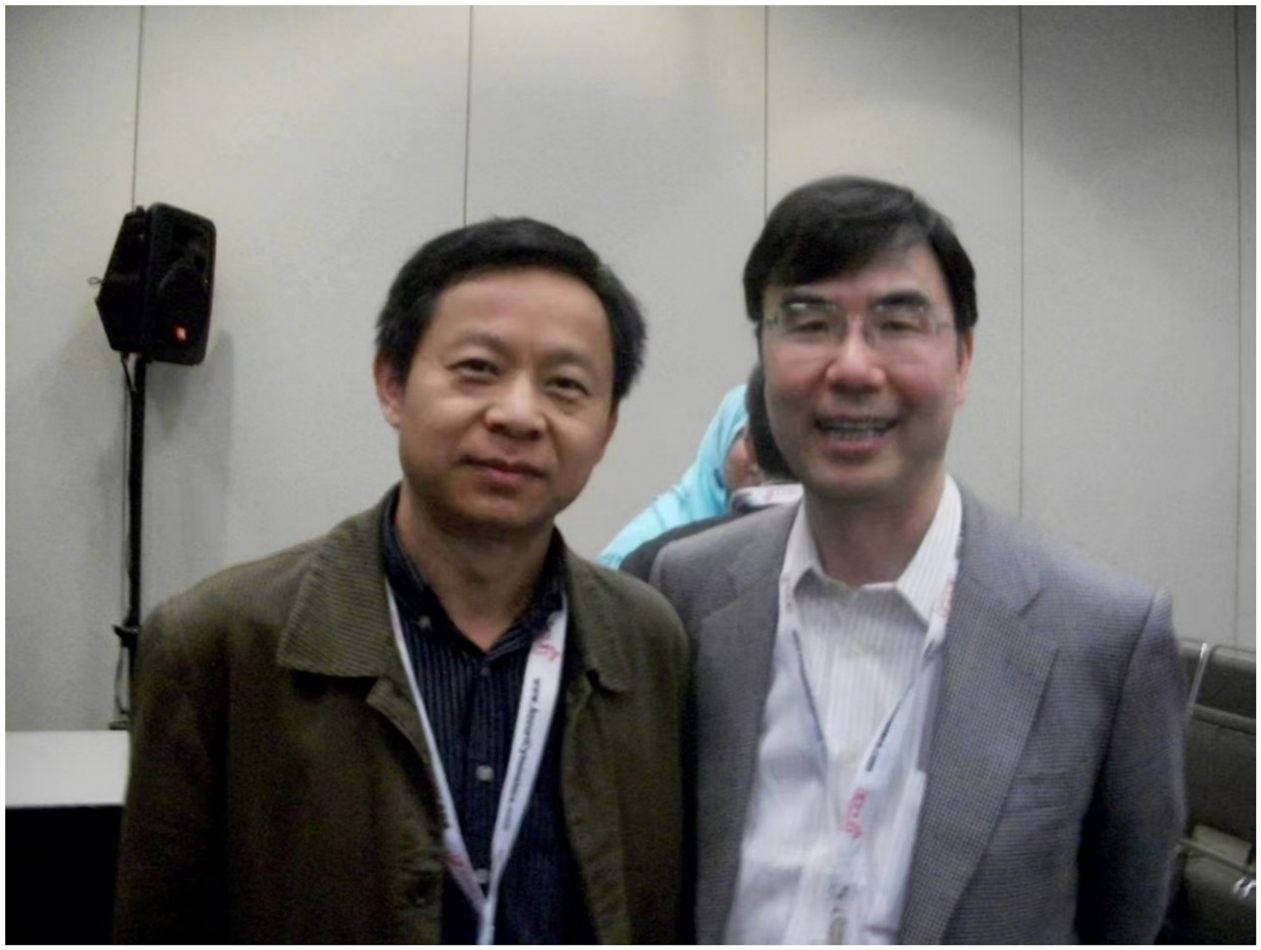
Song Guo Zheng (left) and Shimon Sakaguchi (right) at the 2005 American Association of Immunologists (AAI) conference (Boston) (image provided by Song Guo Zheng)

On October 7, 2025, the Nobel Assembly at Sweden’s Karolinska Institute announced the first Nobel Prize of 2025 — in Physiology or Medicine, as usual. Dr. Sakaguchi finally became a new Nobel laureate due to his creative contribution to the discovery of Treg cells and their role in maintaining immune tolerance [1]. Additionally, two other experts, Mary E. Brunkow and Fred Ramsdell, who have identified *Foxp3* gene mutation and its relationship with immune tolerance breakdown in scurfy mice [2,3]. Olle Kampe, chair of the Nobel Committee, stated that “*their discoveries have been decisive for our understanding of how the immune system functions and why we do not all develop serious autoimmune diseases*”.

As early as the 1960s, Nishizuka and others conducted an interesting experiment. They found that if the thymus was removed from newborn mice on the first to third days after birth (P1–P3), the mice would develop autoimmune inflammatory reactions in various organs. However, if the thymus was removed after P4, this phenomenon did not occur [4,5]. This suggests that certain components or cell populations in the thymus migrate to the periphery of mice by P3, regulating peripheral immune tolerance. Gershon and Kondo, through functional verification experiments, found that thymocytes have immunosuppressive capabilities and can induce infectious immune tolerance [6]. Farmer lab also found that infusing lymphocytes into these thymectomized mice could prevent the occurrence of inflammatory diseases [7]. By 1993, Powrie et al. proposed that a subset of CD4^+^ T cells in the thymus plays an immunosuppressive role in controlling inflammation [8], and Watson lab further suggested that the CD4^+^CD45RB^low^ cell population exerted an important immunosuppressive effect [9].

By 1995, Sakaguchi and his colleagues finally clarified that it was the CD4^+^CD25^+^ cell population in the thymus that plays a decisive role [1]. They verified this cell population through three experiments. (1) Removing the CD25^+^ subset from CD4^+^ cells in the thymus of normal mice and then infusing them into athymic mice could induce various organ inflammations similar to those observed after thymectomy within 3 days of birth. (2) If the CD25^+^ cell population was re-infused into the aforementioned mice, the inflammatory diseases above could be prevented. (3) Using an allogeneic skin transplantation model, it has been further validated that this group of cells is immunosuppressive cells.

In 2001, Brunkow and Ramsdell made an important contribution by discovering that a mutation in a gene called *Foxp3* can cause a systemic fatal autoimmune disease [2,3], and that this mouse disease is very similar to human immune dysregulation, polyendocrinopathy, enteropathy, X-linked (IPEX) Syndrome [10]. However, at that time, people did not know the correlation between this gene and Treg cells.

Three laboratories including Sakaguchi, Rudensky, and Ramsdell in the United States and Japan almost simultaneously discovered that Foxp3 is not only a characteristic marker of Treg cells but also an important molecule for the development and function of Treg cells in 2003 [11–13]. These findings laid an important foundation for promoting research on Treg biology.

Work by Sakaguchi and colleagues in the mid-1990s, particularly the 1995 *J Immunol* article [1], stimulated global interest in whether Treg cells could be harnessed for clinical benefit. Given cells from the thymus cannot be used for cell therapy, it is natural to ask whether a similar differentiation of Treg cells could be achieved like that of T helper 1 (Th1) and Th2 cells. Collaborative efforts from Horwitz and Zheng teams have contributed to the demonstration that Treg cells can be induced in the presence of T-cell receptor stimulation and transforming growth factor-β signaling in 2002 [14], and help establish the concept that Treg cells exert their immunological tolerance through infectious tolerance *in vivo* [15]. These results have been confirmed and highlighted [16–19].

nTreg cells are extremely important; nonetheless, their function is less stable under inflammatory conditions [20]. For instance, Strober lab identified that in the presence of IL-6, nTreg cells can transform into Th17 cells [21]. Although the *Foxp3* gene in iTreg cells is highly methylated [22], *Foxp3* methylation may not be the only factor that determines Treg function. In fact, Sakaguchi lab recently has reported that the blockade of CD28 signal changes the methylation of iTreg cells [23]. In addition, many Chinese scientists also made a great contribution to Treg biology [24,25].

Treg cells have begun to approach clinical application. Blazar and Bluestone have made major contributions to Treg prevention of graft *versus* host disease (GvHD) and organ transplant rejections [26–28]. However, due to the instability of these cells under inflammatory conditions, there are few reports of successful clinical trials in the treatment of autoimmune and inflammatory diseases. Zheng lab has established a method that can potently stabilize nTreg cells under inflammatory conditions. The nTreg cells modified with all-*trans* retinoic acid become highly stable with enhanced functions [29]. These cells are named as the second-generation Treg cells, which have promoted the clinical translation of Treg cells in the treatment of autoimmune diseases. In fact, clinical trials in China for using the second generation of Treg cells are ongoing now.

Sakaguchi and I are currently collaborating on the molecular mechanisms underlying the functional enhancement of Treg cells in the tumor microenvironment, and we have identified some potential molecular targets (data not published). We hope that one day, we can work together to contribute to cancer therapy, as cancers pose a serious threat to the health of people around the world.

## CRediT author statement

**Song Guo Zheng:** Conceptualization, Writing – original draft, Writing – review & editing, Project administration, Funding acquisition. The author has read and approved the final manuscript.
